# Identification of a Novel P190-Derived Breakpoint Peptide Suitable for Peptide Vaccine Therapeutic Approach in Ph+ Acute Lymphoblastic Leukemia Patients

**DOI:** 10.1155/2012/150651

**Published:** 2012-02-15

**Authors:** Micaela Ippoliti, Marzia Defina, Antonella Gozzini, Claudia Baratè, Lara Aprile, Alice Pietrini, Alessandro Gozzetti, Donatella Raspadori, Francesco Lauria, Monica Bocchia

**Affiliations:** ^1^Department of Hematology, University of Siena, 53100 Siena, Italy; ^2^Department of Hematology, University of Florence, 50121 Florence, Italy; ^3^Department of Hematology, University of Pisa, 56126 Pisa, Italy

## Abstract

Ph+ acute lymphoblastic leukemia (Ph+ ALL) is a high-risk acute leukemia with poor prognosis, in which the specific t(9;22)(q34;q11) translocation results in a chimeric bcr-abl (e1a2 breakpoint) and in a 190 KD protein (p190) with constitutive tyrosine kinase activity. The advent of first- and second-generation tyrosine kinase inhibitors (TKIs) improved the short-term outcome of Ph+ ALL patients not eligible for allo-SCT; yet disease recurrence is almost inevitable. Peptides derived from p190-breakpoint area are leukemia-specific antigens that may mediate an antitumor response toward p190+ leukemia cells. We identified one peptide named p190-13 able to induce in vitro peptide-specific CD4+ T cell proliferation in Ph+ ALL patients in complete remission during TKIs. Thus this peptide appears a good candidate for developing an immune target vaccine strategy possibly synergizing with TKIs for remission maintenance.

## 1. Introduction

Philadelphia positive acute lymphoblastic leukemia (Ph+ ALL) is a high-risk, aggressive form of acute leukemia, affecting primarily adults and the elderly. The hallmark of this disease is the presence in all leukemia cells of a reciprocal translocation termed t(9; 22)(q34; q11) resulting in a chimeric bcr-abl (e1a2 breakpoint) fusion gene that encodes a 190 KD protein (p190) with constitutively active tyrosine kinase activity that can alter multiple signaling pathways, contributing to tumor growth and proliferation.

Before the advent of tyrosine kinase inhibitors (TKIs), the outcome of Ph+ ALL patients not eligible for allogeneic stem cell transplant (allo-SCT) was characterized by an extremely poor prognosis, a weak response to most chemotherapy combinations, short remission durations, and poor survival rates. The introduction of imatinib, a selective inhibitor of the ABL tyrosine kinase, has revolutionized the treatment and the outcome of this subset of patients [1]. However, a substantial proportion of imatinib-treated Ph+ ALL patients develop resistance to imatinib. Second-generation TKIs have demonstrated promising efficacy in the treatment of imatinib-resistant Ph+ ALL patients, but despite these results, the relapse rate of Ph+ ALL patients remains very high with an overall survival still unsatisfactory [2]. The persistence of a measurable residual disease at molecular level appears to be the key issue for treatment failure [3–5]. The development of alternative strategies that could selectively target Ph+ ALL cells and synergistically work in combination with TKI may have a crucial impact on disease control and ultimately patients' survival. On this matter, a p190-specific active immune approach like a vaccine could meet these requirements. Due to bcr-abl fusion, the corresponding p190 joint region contains an amino acid sequence unique to the oncoprotein in addition to a novel amino acid, not belonging to either BCR or ABL sequences, created at the exact fusion point. Thus, from an immunologic point of view, peptides derived from p190-breakpoint area are leukemia-specific antigens that may be employed as therapeutic vaccine with the purpose to induce a T cell response toward p190+ leukemia cells. Recently “natural” bcr-abl breakpoint-specific cytotoxic T lymphocytes (CTLs) were found in the bone marrow of Ph+ ALL patients treated with imatinib correlating with a better response to this TKI [6]. These findings suggest a potential activity of the immune system against this lethal disease and the crucial role of p190 itself as target.

In the present work we searched for p190-derived breakpoint peptides suitable for a peptide vaccine approach in vivo. Previously, we have developed a p210-breakpoint derived penta-peptide vaccine for controlling minimal residual disease in Chronic Myeloid Leukemia (CML) patients treated with imatinib [7]. In this setting, we found that the best antileukemia immune response was mediated by CD4+ T cells specific for an HLA class II “size” p210 breakpoint-derived peptide included in the vaccine. p210-breakpoint peptide-specific CD4+ T cells isolated from vaccinated patients were found to be either perforin+ or CD25+/Foxp3+: in both cases they exerted direct cytotoxic activity against a CML cell line [8]. Based on these premises, in our vaccine strategy for Ph+ ALL, we focused our efforts in the search for p190 breakpoint peptides as strong inducers of a peptide-specific CD4+ T cell response. Our results show a promising p190-derived breakpoint peptide suitable for a peptide vaccine therapeutic approach in these patients.

## 2. Material and Methods

### 2.1. p190-Derived Peptide Identification

To pursue our vaccine strategy for Ph+ ALL we investigated the fusion region of p190 in search of novel 25-mer p190 breakpoint peptides with strong HLA class II binding prediction and thus potentially able to induce a strong CD4+ T cell stimulation. The length of 25 amino acids has been chosen as maximum length that should contain all possible HLA class II molecules binding epitopes, usually from 13 to 23 amino acids long, always including the breakpoint and the new amino acid produced at the fusion point. We analysed all 25 possible 25-mer long peptides that include the fusion point (Table 1). We employed Syfpeithi database for MCH ligands and peptides motifs and Bimas database which allow to estimate the ligation strength to a defined HLA type for a sequence of amino acids [9]. The level of binding was compared with our “binding prediction mean database” for HLA class II BCR-ABL-derived peptides already employed in CML patients. Promising peptides will be synthesized on F-MOC solid phase synthesis and purified by HPLC for in vitro use.

### 2.2. CD4+ T Cell Priming In Vitro

In order to evaluate the capability of p190-derived peptides to induce a peptide-specific CD4+ T cell in vitro, we performed a 21-day autologous stimulation assay. Briefly CD4+ T cells freshly isolated from PBMC were cultured for 21 days in 5% AB human serum media while undergoing to 3 rounds of stimulation with autologous CD14+ cells previously isolated and frozen. CD4+ T cells were seeded in 24-well plates at 1 × 10^6^ cell/mL together with CD14+ cells at 0.25 × 10^6^ cell/mL (4 : 1 ratio) and maintained in a humidified incubator with 5% CO_2_ and 95% air at 37°C for 7 days. Each test peptide was added at 20 *μ*g/mL and IL-15 was added at final concentration of 10 ng/mL. After this time CD4+ T cells underwent the second round of stimulation: the cells were collected, counted, and replaced in the same conditions with fresh CD14+ cells, peptides, and IL-15 for other 7 days. The rounds of stimulation were 3. The peptide-specific CD4+ T cell proliferation was measured by standard ^3H^thymidine incorporation assay and expressed by a stimulation index (SI = cpm CD4+ T cells plus test peptides/CD4+ T cell alone or CD4+ T cells plus control peptides). Briefly, CD4+ T cells were collected and incubated at 2 × 10^5^ cells per well for 96 hours and maintained for 4 days in a humidified incubator with 5% CO_2_ and 95% air at 37°C with 20 *μ*g/mL peptide under the following experimental conditions: (1) no peptide, (2) each test peptide alone, and (3) WT1-derived 25 amino acid peptide alone as negative control. The peptide-specific T cell proliferation was considered positive for SI ≥2. The level of p190-peptide specific T cell proliferation was compared with our “stimulation index mean database” for HLA class II BCR-ABL-derived peptides already employed in CML patients. The latter allowed us to identify peptides with good probability to be sufficiently immunogenic and suitable for vaccination studies in vivo.

## 3. Results

Among all 25 possible peptide in the fusion region, we identified three promising 25-mer long p190 fusion peptides: PDNGEGAFHGDAEALQRPVASDFEP (p190-13), DGEGAFHGDAEALQRPVASDFEPQG (p190-15), and EGAFHGDAEALQRPVASDFEPQGLS (p190-17) with strong HLA binding properties for HLA-DRB1*0101, HLA-DRB1*0401, HLA-DRB1*1101, and HLA-DRB1*0301 (DR17). Subsequently p190-13, p190-15, and p190-17 have then been synthesized and purified for in vitro T cell stimulation testing.

In vitro p190-derived peptide CD4+ T cell stimulation was first performed in 5 normal subjects. In each healthy donor p190-13, p190-15, and p190-17 peptides were tested in separated experiments. Only p190-13 peptide was able to induce in all 5 subjects, regardless of their HLA-DR phenotype, a peptide-specific CD4+ T cell proliferation as measured by standard ^3H^thymidine assay, with an SI ranging from 2.0 to 2.7. Similar experiments were performed in 6 Ph+ ALL patients. All patients had previously received a standard induction treatment followed by TKIs therapy and were at least in hematologic remission at the time of in vitro CD4+ T cell experiment. Three of six were receiving dasatinib at 100 mg/day and 3/6 were receiving imatinib at 400 mg/day (see Table 2). Again p190-13 peptide induced peptide-specific CD4+ T cell proliferation in 4/6 Ph+ ALL patients with an SI value of 2.2, 2.0, 2.2, and 2.1, respectively (Table 2). 

## 4. Discussion

The Ph+ ALL is a high-risk acute leukemia with poor prognosis, in which the specific t(9; 22)(q34; q11) translocation results in a chimeric bcr-abl (e1a2 breakpoint) and in a 190 KD protein (p190) with constitutive tyrosine kinase activity. Despite the promising results of TKIs, the relapse rate of Ph+ ALL patients remains very high with an overall survival still unsatisfactory. In Ph+ ALL, leukemic cells could be targeted also by means of an active specific immune response raised against BCR-ABL-derived p190 fusion protein that is indeed a leukemia-specific intracellular antigen. Thus vaccinations with p190-derived peptides could induce in Ph+ ALL patients peptide-specific T cell response that could control or even eradicate minimal residual disease thus reducing the probability of relapse in these patients. In an antitumor vaccine strategy the choice of the appropriate peptide is crucial and we decided to focus our search on longer, HLA class II binder p190-breakpoint peptides, with the intent mainly to prompt a peptide-specific and possibly leukemia-specific CD4+ T cell response.

The present study identified p190-13, a novel p190-derived 25-mer peptide that met these requirements. In fact p190-13 was able to easily induce, in vitro, a peptide-specific CD4+ T cell response in all healthy donors and in 4/6 Ph+ ALL patients tested. Of note, this peptide includes in its sequence all the epitopes for which p190-specific CTLs were naturally found in ALL patients [6] and thus p190-13 could as well mediate a peptide-specific CTLs response in this setting of patients. Regarding the choice of a breakpoint peptide for developing a peptide vaccine strategy in Ph+ ALL we are aware that ALL has a much more instability than CML and thus p190 has more probability to undergo mutations. Even if the latter is true, it has to be underlined that p190 mutations do not usually appear in the breakpoint region which sequence is very stable through all the phases of the disease. The little number of patients studied and the variability of age, chemotherapy induction treatment, and type of TKI do not allow us to speculate further about the quality and intensity of the in vitro peptide-specific immune response observed. However, it is encouraging that 4/6 Ph+ ALL patients were able to mount an in vitro CD4+ T cell response against p190-13 while being treated with imatinib but also with second generation TKIs, dasatinib. The magnitude of p190-derived peptide-specific T cell response, as measured by the stimulation index, was the same in patients and healthy donors and was in the range of what we previously found in CML patients, confirming that ALL patients treated with TKIs maintain a functional and reactive immune system even against a poorly immunogenic self oncoprotein like p190. For all these reasons our peptide could be a good candidate for developing an immune target vaccine strategy possibly synergizing with TKIs for remission maintenance and reducing the probability of relapse in Ph+ ALL patients. These results support the rationale to offer to Ph+ ALL elderly patients or adult patients not eligible for intensive chemotherapy and allo-SCT, a line treatment without chemotherapy, by using a TKI as debulking induction treatment and subsequently by adding a potentially synergic leukemia-specific immune approach such as a p190-derived peptide vaccine.

## 5. Conclusion

In conclusion novel identified p190-13 25-mer peptide is able to induce in vitro a peptide-specific CD4+ T cell response in Ph+ ALL patients. Thus there appears a good candidate for developing an immune target vaccine strategy possibly synergizing with TKIs for remission maintenance. A p190-13 peptide vaccine trial in Ph+ ALL patients in complete hematologic remission during TKIs is currently in planning.

## Figures and Tables

**Table 1:** p190 amino acid sequence (e1a2 breakpoint) and all 25 possible 25-mer peptides that include fusion point.

	BCR ABL
	TIVGVRKTGQIWPNDGEGAFHGDA**E**ALQRPVASDFEPQGLSEAARWNSK
(1)	TIVGVRKTGQIWPNDGEGAFHGDA**E **
(2)	IVGVRKTGQIWPNDGEGAFHGDA**E**A
(3)	VGVRKTGQIWPNDGEGAFHGDA**E**AL
(4)	GVRKTGQIWPNDGEGAFHGDA**E**ALQ
(5)	VRKTGQIWPNDGEGAFHGDA**E**ALQR
(6)	RKTGQIWPNDGEGAFHGDA**E**ALQRP
(7)	KTGQIWPNDGEGAFHGDA**E**ALQRPV
(8)	TGQIWPNDGEGAFHGDA**E**ALQRPVA
(9)	GQIWPNDGEGAFHGDA**E**ALQRPVAS
(10)	QIWPNDGEGAFHGDA**E**ALQRPVASD
(11)	IWPNDGEGAFHGDA**E**ALQRPVASDF
(12)	WPNDGEGAFHGDA**E**ALQRPVASDFE
(13)	PNDGEGAFHGDA**E**ALQRPVASDFEP
(14)	NDGEGAFHGDA**E**ALQRPVASDFEPQ
(15)	DGEGAFHGDA**E**ALQRPVASDFEPQG
(16)	GEGAFHGDA**E**ALQRPVASDFEPQGL
(17)	EGAFHGDA**E**ALQRPVASDFEPQGLS
(18)	GAFHGDA**E**ALQRPVASDFEPQGLSE
(19)	AFHGDA**E**ALQRPVASDFEPQGLSEA
(20)	FHGDA**E**ALQRPVASDFEPQGLSEAA
(21)	HGDA**E**ALQRPVASDFEPQGLSEAAR
(22)	GDA**E**ALQRPVASDFEPQGLSEAARW
(23)	DA**E**ALQRPVASDFEPQGLSEAARWN
(24)	A**E**ALQRPVASDFEPQGLSEAARWNS
(25)	**E**ALQRPVASDFEPQGLSEAARWNSK

**Table 2:** p190-13 peptide-specific CD4+ T cell proliferation obtained in 5 healthy donor (HD) and 6 Ph+ ALL patients (HD: healthy donor; SI: stimulation index; HR: hematologic remission).

	Age, sex	Disease stage	Current treatment	p190-13 CD4+ T cell SI
HD 1	58 M	—	—	2.0
HD 2	43 F	—	—	2.5
HD 3	55 M	—	—	2.3
HD 4	34 M	—	—	2.7
HD 5	52 F	—	—	2.3
Ph+ ALL 1	73 M	HR	dasatinib	1.0
Ph+ ALL 2	69 F	HR	dasatinib	1.0
Ph+ ALL 3	57 F	HR	dasatinib	2.2
Ph+ ALL 4	43 M	HR	imatinib	2.0
Ph+ ALL 5	64 F	HR	imatinib	2.2
Ph+ ALL 6	72 F	HR	imatinib	2.1
